# Development of Zeolite Imidazole Framework-Based Adsorbent for Effective Microextraction and Preconcentration of Histamine in Food Samples

**DOI:** 10.3390/foods13162564

**Published:** 2024-08-16

**Authors:** Abdullah Khalid Omer, Hossein Tajik, Rahim Molaei, Mehran Moradi

**Affiliations:** Department of Food Hygiene and Quality Control, Faculty of Veterinary Medicine, Urmia University, Urmia 1177, Iran; abdullah78@yahoo.com (A.K.O.); h.tajik@urmia.ac.ir (H.T.); rahimmolaie@yahoo.com (R.M.)

**Keywords:** zeolite imidazole frameworks, histamine, preconcentration, adsorbent, microextraction, foods

## Abstract

This study is the first to focus on the preconcentration and determination of histamine (HIS) in food samples using zeolite imidazole frameworks (ZIFs) on a solid-phase microextraction (SPME) platform. ZIF was developed on a polypropylene hollow fiber (PPHF) substrate (ZIF@PPHF) and characterized. The extraction performance was optimized by adjusting several parameters, including pH, contact time for adsorption, and desorption conditions. Under the optimized conditions, a wide linear dynamic range (0.05–250 mg/L) with high R^2^ values (0.9989), low limit of detection (0.019 mg/L), and low limit of quantification (0.050 mg/L) were determined as analytical figures of merit. Additionally, a reusability study confirmed that ZIF@PPHF preconcentrated 83% of the HIS up to the fourth cycle. The developed method was used to preconcentrate HIS in fish and cheese samples. The spiked real samples confirmed the validity and accuracy of this method. The percentage mean recoveries ± relative standard deviation (% RSD, *n* = 3) at the concentration levels of 5, 10, and 50 mg/L of HIS and the sample amount of 5 g for intra- and inter days ranged from 97 ± 1.10 to 102.80 ± 0.90 and from 96.40 ± 1.82 to 103.40 ± 0.79, respectively. The results suggest that the analytical method validation parameters were acceptable, indicating the repeatability and sensitivity of the method.

## 1. Introduction

Biogenic amines (BAs) are a group of potentially harmful compounds found in foods. They are naturally occurring amines generated by enzymatic decarboxylation of endogenous amino acids [1,2] or by amination and transamination of aldehydes and ketones [3,4,5]. Histamine (HIS) is a member of the family of BAs and has essential physiological functions in humans. It can be found in a wide range of foods and drinks, including fish and fish products [6,7], meat and meat products [8], fermented sausage [9], dairy products [10], and plant and fermented foods [11,12], as well as in beverages [13,14] and other fermented and non-fermented products.

HIS contamination poses an essential challenge for food safety regulators, producers, and consumers owing to its association with foodborne illness, gut disorders, and allergic reactions [15,16,17]. Accordingly, various regulatory organizations have set certain thresholds for HIS levels in different food items to protect public health and prevent incidents of HIS poisoning [18]. Various approaches, including colorimetric, fluorometric, and immunoassay techniques, have been suggested as suitable options for measuring HIS, and most analytical methods rely on chromatographic techniques [19]. However, the complexity of food matrices is a significant problem in quantifying HIS in food [20]. Therefore, the development of reliable and specific analytical methods of evaluating HIS levels in the presence of various interfering compounds is crucial.

Analysts have suggested the use of cleanup and preconcentration procedures. Compared to costly and time-consuming methods that use a lot of chemicals and generate waste, microextraction methods such as solid-phase microextraction [21], dispersive liquid-phase extraction [22], dispersive solid-phase extraction [23], magnetic solid-phase extraction [24], molecularly imprinted polymers [25], and stir-bar sorptive extraction [26] are preferred. Related to the above criteria, Molaei et al. [27] validated using mesoporous silica-coated magnetic nanoparticles as adsorbents to extract HIS and other common BAs in dairy products. Recently, Jayasinghe et al. [28] reviewed several microextraction strategies for extracting HIS from food products. These strategies include solid- and liquid-based microextraction. These processes are small-scale methods for preparing samples for HIS separation and pre-concentration. Furthermore, Alizadeh et al. [29] successfully applied headspace SPME combined with ion mobility spectrometry to measure HIS at low concentrations in various canned fish samples, without using derivatization processes [29].

Metal–organic frameworks (MOFs) are a new type of organic porous material that self-assembles from organic ligands and metal ions to form a network structure [30]. The structural arrangement of metal clusters and organic ligands and their binding styles control the characteristics and structure of MOFs [31,32]. MOFs have demonstrated their practicality in various applications such as storage, separation, sensing, catalysis, and drug delivery. These applications benefit from the increased versatility of the multiple MOF types and synthetic processes. Owing to their unique three-dimensional structures, zeolite imidazole frameworks (ZIFs), a subclass of MOFs, have shown promising results in many fields [33]. ZIFs have attracted the interest of many scientists because they combine the features of zeolites and MOFs. ZIFs are based on the typical cages and channels seen in zeolites but are more accessible to structural changes. ZIFs are preferable to other MOFs in terms of mechanical and chemical stability because of the strength of their metal–nitrogen connections [34,35,36].

The unique characteristics and structures of ZIFs and their large surface areas provide many active sites for adsorption, pre-concentration, and extraction applications. These properties allow efficient interactions with the target molecules, thus improving the preconcentration and extraction efficiency. For example, the Fe_3_O_4_@ZIF-8 adsorbent, with a ˃470 m^2^/g surface area, has shown remarkable efficiency in extracting doxorubicin from aqueous solutions [37]. Additionally, ZIF-8 has recently been used [38] as an efficient sorbent for extraction, applied as a solid-phase extraction (SPE) sorbent, and used as a preconcentration of several benzomercaptans, including “2-mercaptobenzothiazole, 2-mercaptobenzoxazole, and 2-mercapto-6-nitrobenzothiazole”, from environmental water and soil samples. Recently, ZIF-67 was coupled with a magnetic porous porphyrin organic polymer to preconcentrate and enrich neonicotinoid pesticides in river water [39].

In contrast, food samples with a complex matrix have been subjected to improved sample analysis techniques using the in situ growth of ZIFs on layered double hydroxides (LDHs) to prepare porous nanocomposites. This nanoporous composite was prepared by the in situ growth of ZIF-8 on the Zn-Al LDH surface (Zn-Al LDH/ZIF-8) and applied for stir-bar sorptive extraction and the detection of trace amounts of benzylpenicillin in milk samples [40]. Moreover, a new type of substance (Zn-BTEC@ZIF-8) consisting of two different MOFs arranged in a heterostructured mode was produced and designed for HIS detection in seafood with a high sensitivity and low LOD of 1.458 mg/L [41].

This study is the first to explore the use of a ZIF@PPHF adsorbent to extract and concentrate HIS from cheese and fish. The prepared adsorbent was subjected to SPME, which enabled the method to achieve lower limits of detection and quantification. This work also aimed to establish a precise and specific analytical approach for measuring the amount of HIS using a ZIF adsorbent to preconcentrate HIS and extract it via SPME, along with high-performance liquid chromatography (HPLC), which was verified by evaluating its accuracy, precision, linearity, limit of detection (LOD), and limit of quantification (LOQ).

## 2. Materials and Methods

### 2.1. Chemicals and Reagents

Histamine dihydrochloride (HIS), benzoyl chloride (BC), tetraethyl orthosilicate (TEOS), and zinc nitrate hexahydrate solution (Zn(NO_3_)_2_·6H_2_O) were obtained from Sigma-Aldrich (Saint Louis, MO, USA). Ammonia solutions with a concentration of 25% (*w*/*v*), high-purity sodium silicate, acetonitrile, methanol, 2-propanol, acetone, and ethanol (of HPLC grade) were obtained from Merck (Darmstadt, Germany). Sodium hydroxide (NaOH), sodium chloride (NaCl), and hydrochloric acid (HCl) were purchased from Scharlau (Barcelona, Spain). Ultrapure water (DI) was used as the primary water source in all experiments. The Accurel Q3/2 polypropylene hollow fiber was purchased from Membrana GmbH (Wuppertal, Germany).

### 2.2. Preparation of Standard Solution and Derivatization Steps

HIS standard solution with a concentration of 1000 mg/L was prepared in 0.1 M HCl and stored in glass-stoppered bottles at a controlled temperature of 4 ± 1 °C. Working standard solutions were prepared daily by diluting the HIS stock solutions in DI water to obtain parts with desired concentrations.

With some modifications, derivatization of the standard solution was performed according to the method described by Wang et al. [42]. A working standard solution (10 mL) was placed in a glass tube, and 2 mL of 2 M NaOH solution was added to obtain a pH of 12. The as-prepared adsorbents were then immersed in a standard solution and stirred continuously for the desired time. The ZIF adsorbent was then removed and placed in 0.5 mL of a desorption solvent (HPLC-grade methanol) for 2 min. Subsequently, the adsorbent was removed from the desorption solvent, and 20 µL of BC was added to the solvent for derivatization and mixed thoroughly for 30 s. Finally, the mixture was incubated at room temperature for 30 min to complete derivatization. Finally, the HPLC analysis was performed by injecting 10 µL of the treated solution.

### 2.3. Chromatographic Conditions

HPLC analysis was performed using a Shimadzu Prominence-i LC-2030C Plus device equipped with a UV–Vis detector. Chromatographic separation was performed using Nucleoshell RP 18 plus a reversed-phase column (particle size; 2.7 µm, internal diameter; 3.0 mm, length: 150 mm; Macherey-Nagel). In this study, a new column was installed and equilibrated with the mobile phase following the manufacturer’s instructions. First, a new HPLC column was flushed at 0.1–0.5 mL/min for 60 min with 100% mobile phase, which equilibrates the column and mobile phase. The flow rate was then gradually increased to the desired operational flow rate (1.0 mL/min), and the column pressure was monitored to ensure that the peak shape, retention time, and baseline stability met the standards. The mobile phase was a 40:60 mixture of acetonitrile and DI water. The flow rate was set to 1 mL/min, using a 10 μL injection volume and detection at 198 nm at room temperature. After each run, the column was reconditioned with mobile phase for 8.0 min. The samples were eluted using a consistent isocratic method [43,44]. The experiments were conducted in triplicate, and the mean values were recorded. LabSolutions software (Version 5.92) was used for both instrumentation analysis and control.

### 2.4. ZIF Synthesis and Characterization

**Figure 1:**
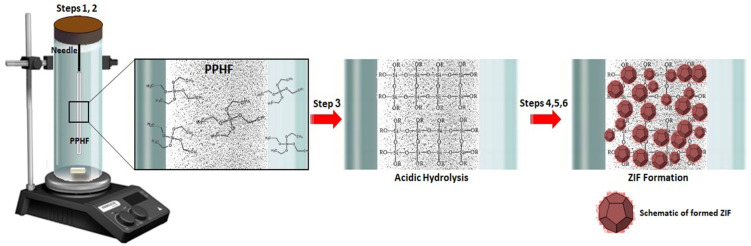
Schematic diagram illustrating the synthesis procedure for zeolite imidazole framework-based adsorbent on a polypropylene hollow fiber (PPHF).

The synthesis and manufacture of the ZIF were conducted using the following procedure: (1) an iron needle measuring 1.5 cm in length was coated with 0.5 cm of polypropylene hollow fiber (PPHF). Subsequently, the needle was placed and fixed in a tube containing 10 mL ammonia solution; (2) 4 mL of tetraethyl orthosilicate (TEOS) was dropped into a tube containing an iron needle covered with PPHF using a burette and stirred constantly at room temperature for 12 h to complete the reaction; (3) after that, the needle was removed and immersed in HCl (pH 5) for 12 h without stirring; (4) the needle was then withdrawn, submerged in a zinc nitrate hexahydrate (0.01 M) solution, and subjected to gentle agitation for 12 h; (5) the needle was removed and washed with water and ethanol; and (6) in the last step, the needle was immersed in a 2-methylimidazole solution (0.10 M, in methanol) at room temperature overnight. The needle was then removed and analyzed (Figure 1). Different techniques, including field emission scanning electron microscopy (FESEM; SIGMA VP-500; Zeiss, Jena, Germany) and Brunauer–Emmett–Teller (BET; Belsorp II apparatus, Osaka, Japan) were used to characterize the synthesized platform. The textural features and surface area of the modified PPHF were also evaluated using nitrogen adsorption isotherms. Attenuated total reflectance–Fourier transform infrared (ATR-FTIR) (Thermo Electron, Nexus^®^ 670, Madison, WI, USA) (450–4000 cm^−1^, 4 cm^−1^) spectra were employed to provide a comprehensive understanding of the in-site growth of ZIF on PPHF and the surface functional groups of the as-prepared absorbent.

### 2.5. Optimization Conditions of the Analytical Parameters

Several experimental parameters, including pH, adsorption contact time, and desorption conditions (elution solvent type) were optimized using one-factor-at-a-time. In addition, the food matrices and the reusability of the adsorbents were thoroughly studied to verify and improve the sensitivity of the method. It should be noted that every experiment was conducted three times, and the optimal conditions were identified.

The reusability of the prepared adsorbent was assessed through multiple adsorption–desorption cycles. After each cycle, the adsorbent was rinsed twice with deionized water and air-dried at room temperature before reuse. Serial analyses were conducted with 10 mL of HIS standard working solution at a concentration of 10 mg/L throughout the six reuse cycles to assess the reusability and adsorption efficiency (*R* %) of the adsorbent, which was calculated using Equation (1) [45].

(1) $$\%\;R=\frac{C_{i}-C_{f}}{C_{i}}\times 100$$

where *C_i_* is the initial concentration of the HIS working solution and *C_f_* is the final concentration of the HIS solution throughout all six reuse cycles.

### 2.6. Extraction, Preparation, Preconcentration, and Derivatization Procedure for HIS Detection in Fish and Cheese Samples

**Figure 2:**
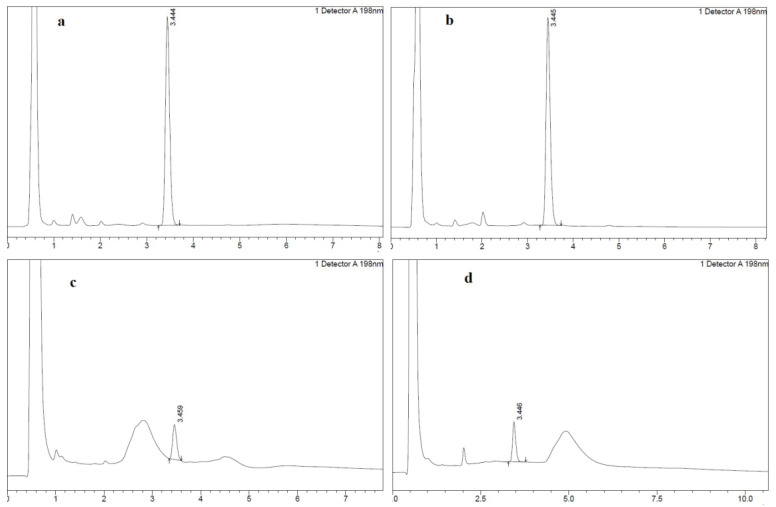
HPLC chromatograms of (**a**) standard solution without adsorbent, (**b**) standard solution using prepared ZIF@PPHF adsorbent, (**c**) fish samples using prepared ZIF@PPHF adsorbent, and (**d**) cheese samples using prepared ZIF@PPHF adsorbent. The chromatogram clearly illustrates that the total runtime of the analysis was 8 min. The retention time varied from 3.444 min to 3.459 min.

HIS extraction, preconcentration, and derivatization procedures were conducted following the method described by Molaei et al. [27], with slight modifications. High-fat fish (flathead mullet; *Mugil cephlus*; ~11%, low-fat fish (common carp; *Cyprinus carpio*; ~2%), high-fat cheese (feta~18%), and low-fat cheese (cheddar~6%) samples were weighed separately (5 g) and homogenized with 5 mL HClO_4_ (5% *v*/*v*). Homogenates were refrigerated (4 ± 2 °C) for 1 h. The mixture was centrifuged at 4000× *g* for 10 min at approximately 4 °C and then filtered through filter paper with 20 μm pores to collect the extract in a volumetric flask. The remaining solids were extracted using the same procedure and the extracts were filtered through the same flask. The volume was completed with DI water, neutralized (pH > 6) with 2 M NaOH, and stored in an ice bath (0 ± 2 °C) for 20 min. Subsequently, the pH of the mixture was adjusted to 12 by the addition of 2 M NaOH and vortexing for 30 s. Afterward, an aliquot of the sample extraction solution (10 mL) was filtered into a tube using a disposable syringe filter “FilterBio^®^ Nylon Syringe Filter, 33 mm diameter, 0.45 µm pore size”, and the prepared adsorbents were dipped in a sample extraction solution and stirred continuously for 7 min. In the next step, the prepared ZIF adsorbent was removed from the extraction solution and immersed in a 0.5 mL desorption solvent (HPLC-grade methanol) for 2 min. The prepared adsorbent was then removed from the desorption solvent and 20 µL of BC was added to the solvent for derivatization and mixed thoroughly for 30 s. Subsequently, the mixture was left at ambient temperature for 30 min to allow the completion of the derivatization reaction. The extracted solution was subjected to HPLC analysis by injecting 10 µL extract. The analysis had a total run time of 8 min, and the retention time ranged between 3.444 and 3.459 min, as shown in the HPLC chromatograms (Figure 2). The selection of variables and materials for this test was based on the optimized results obtained in the previous stage, as detailed in Section 3.

The matrix effect (*ME*) was assessed by comparing the peak areas of real fish and cheese samples spiked with HIS standards at concentrations of 5, 10, and 50 mg/L against the peak areas of the same HIS standards at identical concentrations in the absence of the sample matrix. *ME* can be determined by comparing the response of an analyte in a standard solution to that in a spiked sample and can be calculated using Equation (2) [46].

(2) $$\%\;ME=\frac{B}{A}\times 100$$

where *B* is the peak area of the analyte in the spiked sample, and *A* is the peak area of the analyte in the standard solution.

The enrichment factor (*EF*) of the method was calculated using Equation (3) [47].

(3) $$EF=\frac{V_{s\;}}{V_{el}}\times R\;\%$$

where *V_s_* is the sample volume, *V_el_* is the elution volume, and *R* % is the quantitative percentage recovery.

### 2.7. Method Validation

The performance of the proposed method was validated using various measures, including accuracy (recovery rate), precision (intra- and inter-day), linear dynamic range (LDR), determination coefficient (R^2^), LOD, and LOQ (Table 1). The proposed method was validated using spiked real fish and cheese samples. Moreover, accuracy (recovery rate) and intra- and inter-assay precision (percent relative standard deviations, %RSD) were also evaluated. Three replicates of 5 g samples were used to assess accuracy and the %RSD inter-day and intra-day precision, which spiked with three concentration levels of 5, 10, and 50 mg/L of HIS (data from two real food samples—fish and cheese). Analyses were conducted 72 h apart using the same samples and concentrations as those used to assess intra-day precision.

## 3. Results and Discussion

### 3.1. Characterization of ZIF@PPHF Adsorbent

#### 3.1.1. FESEM and BET

**Figure 3:**
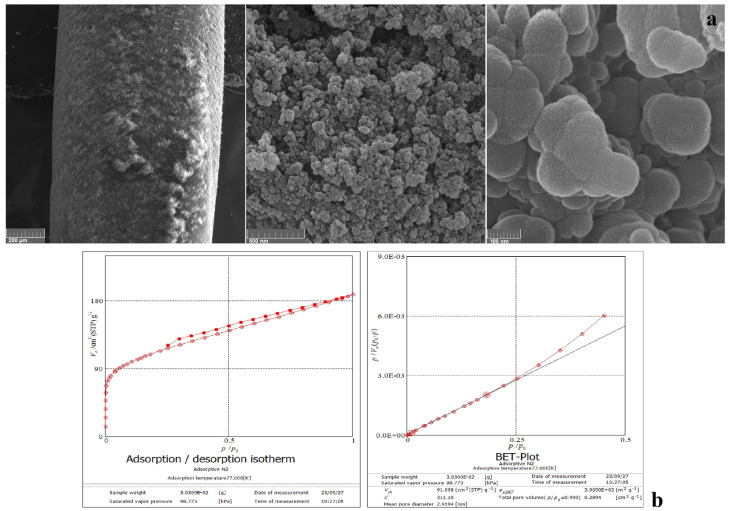
FESEM micrograph of the prepared ZIF@PPHF adsorbent at different magnifications (**a**), (N_2_) adsorption–desorption isotherms, and BET plot (**b**).

To determine the size and morphology of the ZIF structure grown on the PPHF substrate, FESEM micrographs were captured at various magnifications (Figure 3a). As can be seen, the size of the nano-crystals is about 100 nm, and all particles are semi-spherical, with some protrusions. According to IUPAC categorization, the gas (N_2_) adsorption–desorption curves of the prepared fiber (Figure 3b) showed behaviors very similar to type IV hysteresis loop isotherms, which are specific to mesoporous materials [48]. Moreover, the Brunauer–Emmett–Teller (BET) plot was based on the adsorption–desorption isotherms of liquid N_2_ at 77 K), and the surface area of the fiber was approximately 396.5 m^2^/g (Figure 3b).

#### 3.1.2. ATR-FTIR Analysis

**Figure 4:**
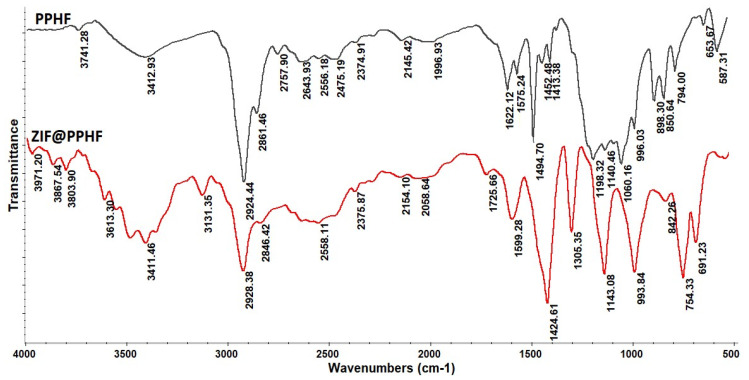
ATR-FTIR spectra of PPHF and the prepared adsorbent (ZIF@PPHH).

As depicted in the PPHF spectrum (Figure 4), the characteristic absorption bands centered at 2924 and 2861 cm^−1^ and the peaks observed at approximately 1500–1400 cm^−1^ can be assigned to the stretching vibration modes of the-CH_2_-, -CH-, and -CH_3_ groups of the polypropylene material. In addition, the intertwined peaks around 1200–1000 cm^−1^ can be attributed to C–C asymmetric stretching, CH_3_ asymmetric rocking, and C–H wagging vibrations, whereas the absorptions at 898 and 850 cm^−1^ can be attributed to CH_2_ rocking vibrations [49]. In contrast, in the ATR-FTIR spectrum of the designed absorbent (Figure 4), all the characteristic peaks related to the C-H stretching of the aromatic (at 3131 cm^−1^) and aliphatic (at 2928 cm^−1^) imidazole rings; the absorption band of the C–N bond (peaks at 1424–1305 cm^−1^) and out-of-plane (1143 and 993 cm^−1^) and in-plane (754–691 cm^−1^) bending of the imidazole ring are related to the on-site growth of ZIF on PPHF [50].

### 3.2. Optimization Analytical Parameters

#### 3.2.1. pH

**Figure 5:**
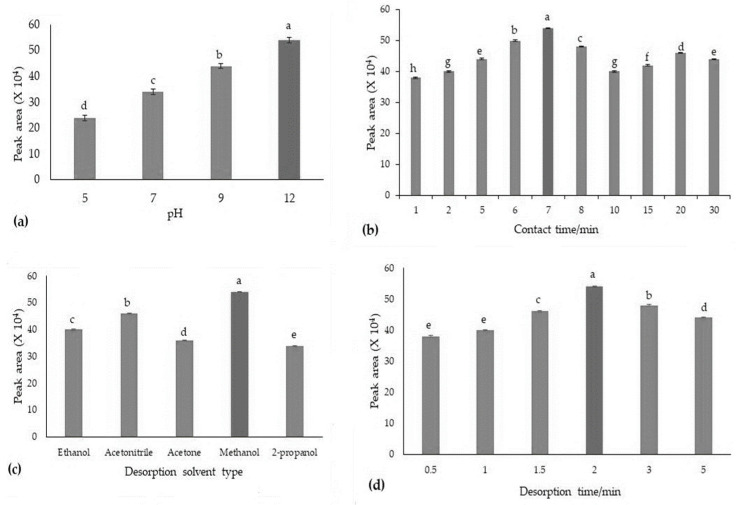
The optimization conditions for the prepared adsorbent in the extraction procedure, based on a one-factor-at-a-time approach, involved optimizing all relevant parameters using 5.0 mL of a working standard solution at a concentration of 10 mg/L, with the volume of elution solvent fixed at 0.5 mL. The optimization factors were the pH of the solution (pH 5, 7, 9, and ≥12) (**a**), adsorption contact time (1.0–30 min) (**b**), desorption solvent type (**c**), and desorption solvent time (0.5–5.0 min) (**d**). Values were considered statistically significant at *p* < 0.05, and values with different letters indicate significant differences.

The solution pH has an important effect on the chemical reactions caused by contaminants, ion competition for binding sites, and functional group activity (surface charge) in the adsorbent [51]. Therefore, the effects of various pH levels (5, 7, 9, and ≥12) on the adsorption capacity of ZIF@PPHF adsorbents and the optimal pH conditions for achieving the highest adsorption capacity were investigated. The data demonstrated a clear trend, with the adsorption capacity increasing as the pH of the solution increased and reaching the optimum adsorption rate at pH ≥ 12 (Figure 5a). Similar to our study, Nasir et al. [52] successfully synthesized ZIF-L at ambient temperature for arsenic adsorption. They found that alkaline conditions at pH 10 resulted in the highest adsorption capacity. Similarly, the highest amount of 1-naphthol adsorbed by ZIF-67 was observed in a solution with a pH of 10 [53].

As previously stated, ZIFs exhibit a remarkable porous structure characterized by uniform pore sizes, making them attractive for various applications including analytical pretreatment and adsorption [54,55,56]. Owing to their large surface area and highly porous nature, ZIFs have many sites for absorbing compounds. Ideally, the analyte and adsorbent should have opposite surface charges or polarities to enable efficient interactions [57,58]. These factors ultimately determine the surface interaction between the analyte and adsorbent as well as the optimal extraction efficiency. The pores are influenced by the interplay between the acid and base interactions, electrostatic interactions, π-bond stacking, coordination interactions, and hydrogen bonding. These factors facilitate the selective adsorption of molecules that fit perfectly within the pores, while excluding those that are either too large or too small [59].

One way to explain why analytes stick to the solid phase is by examining the pH value called the isoelectric point (pHpzc). At this pH, the surface has no net charge because the positive and negative charges are balanced. Above this pH, the surface is negatively charged, whereas below it, the surface is positively charged [60]. In this study, the pH of the isoelectric point or point of zero charge (PZC), pHpzc, was determined using the salt addition technique [61], and a 7.6 value was obtained. Within the pH range < 7.6, ZIFs have a net positive surface charge, causing them to attract anions (species with a negative charge). Conversely, at pH values greater than 7.6, ZIFs attract cations (positively charged species). Furthermore, electrostatic contact does not contribute to the absorption if the adsorbent is not in an ionic state (beyond the acid dissociation constant). Therefore, the pH of the solution significantly influences the electrostatic attraction strength. Interactions between the π and π systems were also observed in ZIF adsorption because of the benzene rings. The rings mentioned are areas with abundant electrons that cause a stacking effect between the acceptor and donor molecules and other aromatic adsorbate species [62].

#### 3.2.2. Adsorption Contact Time

An optimal contact time is crucial for achieving maximum adsorption efficiency and providing an ideal interaction between the analyte and adsorbent. To assess its impact, we investigated the effect of the adsorption time by testing various time intervals ranging from 1 to 30 min. Our results reveal a clear pattern: the adsorption of analytes increased steadily for up to 7 min before leveling off (Figure 5b). As a result, we concluded that a 7 min period represents the most effective adsorption time for all subsequent studies. This highlights the importance of optimizing the adsorption period for optimal HIS extraction using the ZIF@PPHF adsorbents (Figure 5b). The high surface area and porous structure of the ZIF@PPHF adsorbents facilitated rapid mass transfer and diffusion of HIS to active adsorption sites, leading to efficient capture within a shorter duration. This helps maintain the effectiveness and ability of the adsorbent to continuously absorb HIS. By precisely adjusting the contact period to a specific range of 7 min, the adsorption process can be accelerated, operational costs can be reduced, and overall process efficiency can be improved.

#### 3.2.3. Desorption Solvent Type

Choosing the most suitable desorption solvent is crucial in methodological design because it ensures the complete and quantitative release of the analyte. NaOH, methanol, and ethanol are the most suitable and effective eluents for adsorbates from ZIF-based adsorbents [62]. In this study, several eluents (i.e., desorption solvents) with various polarities, including acetonitrile, 2-propanol, methanol, ethanol, and acetone, were tested for their ability to efficiently desorb HIS from adsorbents during extraction and preconcentration. Among these solvents, methanol demonstrated optimal performance, exhibiting ideal desorption rate conditions and superior extraction efficiency (Figure 5c). Additionally, acetonitrile and methanol were the most polar, whereas 2-propanol was the least polar solvent. Given that HIS has a larger structure with multiple polar functional groups contributing to its overall dipole moment, the matching polarities significantly enhanced adsorption efficiency when methanol and acetonitrile were used. Therefore, methanol was selected as the optimal elution solvent in this study.

#### 3.2.4. Desorption Time

Finding the optimal desorption duration is crucial for maximizing the efficiency of releasing or desorbing HIS from the prepared adsorbent. This study conducted to determine the optimal desorption period, ranging from 1 to 30 min, offers useful information regarding the speed of the desorption process and aids in establishing guidelines for efficient HIS recovery when applied as an adsorbent. The consistent desorption efficiency observed across multiple desorption periods indicates that the desorption process was likely fast and effective under the conditions tested. However, it is important to recognize that, even though there were no significant differences, it is recommended that the desorption time is limited to 2 min for efficient HIS desorption. Additionally, optimizing the desorption duration to a specific range of 2 min simplifies the process, reduces operational costs, and enhances the overall appeal of the procedure (Figure 5d).

#### 3.2.5. Extractant (Desorption Solvent) Volume

An in-depth study was carried out to optimize the efficiency of various desorption solvent volumes (0.5–2.0 mL) for the extraction of HIS. The results indicated that 0.5 mL was sufficient to extract HIS and produced the best extraction rate. Thus, methanol (0.5 mL) was selected as the optimal volume for desorption.

#### 3.2.6. Reusability

**Figure 6:**
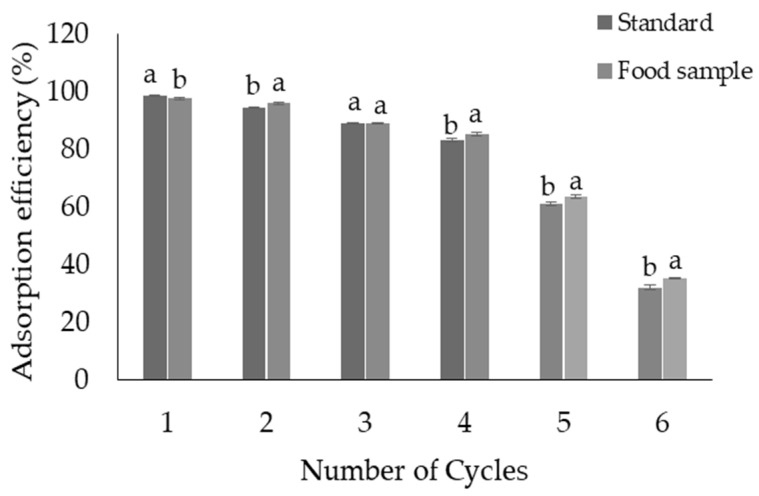
Evaluation of the adsorbent adsorption capacity (%) and reusability over six reuse cycles. The experimental conditions included a working standard solution with a concentration of 10 mg/L and a volume of 10 mL and a typical fish sample with a concentration of 10 mg/kg and a volume of 10 mL (the concentration of histamine in the fish sample was 3.7 mg/kg; to reach the same concentration as that of the standard, 6.3 mg/kg of a standard histamine solution was added). The pH was set to 12, the contact period was 7 min, and the desorption volume was 0.5 mL. Values were considered statistically significant at *p* < 0.05, and values with different letters indicate significant differences.

The reusability investigation confirmed that the ZIF@PPHF adsorbent could effectively preconcentrate 83% of the HIS for up to four cycles (Figure 6). This indicates that the adsorbent demonstrates strong evidence of high reusability. However, as the number of cycles increased, the adsorption capacity gradually declined, with a significant and rapid decrease occurring during the fifth and sixth cycles. The significant decrease in the ability of the ZIF adsorbent to adsorb HIS after six reuse cycles may be attributed to factors affecting its structural integrity, surface chemistry, and overall effectiveness. Repeated cycling can also reduce the surface area and pore size of ZIF. ZIF pores can capture certain adsorbates, particularly those with strong binding affinities such as HIS. The adsorption capacity of the ZIF is primarily attributed to the functional groups present on its surface or within its pores. Repetitive cycles can lead to chemical changes in these groups, reducing their effectiveness in capturing the targeted molecules. In a study conducted by Abdi et al. [63], a dye desorption test was performed using ZIF-8 as the adsorbent. The recovered adsorbents underwent a series of washing steps with distilled water and were dried overnight under a vacuum at 50 °C. The results indicated that the APTES@ZIF-8 adsorbent gradually decreased the dye removal efficiency after four cycles [63]. Moreover, a recent review by Ighalo et al. [62] revealed that a regenerated ZIF-based adsorbent exhibited excellent reusability, with an overall retention rate above 70% after 3–4 cycles [62]. Similarly, an earlier study reported that the Fe_3_O_4_@ZIF-8 adsorbent could be reused up to six times [37].

### 3.3. HIS Detection in Fish and Cheese Samples

#### 3.3.1. Matrix Effect

The presence of MEs—specifically those caused by substances such as fats, proteins, salts, mineral elements, and other BAs found in food samples—can substantially affect the rate and quantity of BA extraction [64,65]. Comprehending and resolving these matrix interferences is essential for precise and reliable analysis of target analytes in food products. A cleanup step is usually necessary when analyzing complicated matrices [66]. HIS detection in food is challenging because of the low analyte concentration and complexity of the sample matrix. Enhancing the performance of analytical techniques and improving detection and quantification limits frequently necessitate sample preparation, purification, and analyte concentration. This process aims to reduce interference from complex food matrices by eliminating substances that disrupt or selectively extract the analytes [20]. Using Equation (2), the ME is expressed as a percentage, showing how much the sample matrix affects the analyte response compared to the standard solution. The ME values for HIS range from 93.63% to 104.06% (Table 1), suggesting that effective MEs do not impact this analytical method [42].

#### 3.3.2. Enrichment Factor (*EF*)

To evaluate the possibility of enriching low concentrations of HIs from large sample volumes, 5 mL of 10 mg/L HIS extract was diluted to 10, 20, 30, 60, 75, and 100 mL. Quantitative recovery (99.6%) was obtained in 60 mL. As previously described, the final amount of analyte was 0.5 mL; therefore, the theoretical enrichment factor was 119, which verifies the feasibility of determining HIS at different concentrations.

### 3.4. Method Validation and Quality Control

The validity of the suggested method in this study was assessed through the various values presented in Table 1. Calibration curves were obtained under the optimized conditions, and the tests showed a wide LDR (0.05–250 mg/L) with high R^2^ values (0.9989) as well as low LODs (0.019 mg/L) and LOQs (0.050 mg/L), which were calculated based on signal-to-noise ratios of 3 and 10, respectively. High R^2^ values indicate strong linearity within the calibration curves, whereas wide LDR values allow analysts to accurately determine analyte concentrations over a wide range. The accuracy of HIS measurement using the ZIF@PPHF adsorbent was assessed in fish and cheese samples spiked with HIS standards at 5, 10, and 50 mg/L. The relative recovery percentage (*RR*, %) was calculated using Equation (4) [67].

(4) $$\%\;RR=\frac{C_{spiked\;sample}-C_{unspiked\;sample}}{C_{added}}\times 100$$

A spiked sample was used to validate the proposed methodology, ensuring the recovery of the HIS levels in food samples. Additionally, it was used to evaluate the effect of the matrix on the extraction efficiency of the method and to assess the repeatability and reproducibility of the approach. Each spiked sample was extracted and derivatized using the same procedure as that for the non-spiked samples. The percentage mean recoveries ± relative standard deviation (% RSD, *n* = 3) (both intra- and inter-day) at concentration levels of 5, 10, and 50 mg/L of HIS and using 5 g samples ranged from (97 ± 1.10 to 102.80 ± 0.90) and from (96.40 ± 1.82 to 103.40 ± 0.79), respectively, for the spiked real samples (Table 1). These findings indicate positive results regarding the validation parameters of the analytical method, underscoring its strong reproducibility and sensitivity. They also underscore the efficacy of this method in accurately quantifying and monitoring histamine levels in fish and cheese samples.

### 3.5. Comparative Analysis of the ZIF@PPHF-Based SPME Method with Other SPME Methods

Compared to previously published methods (Table 2), the current method offers several advantages, such as a lower detection limit, shorter adsorption contact time, and reduced desorption time. In previous studies conducted by Molaei et al. [27] and Hashemi et al. [68], adsorbents were developed to measure histamine levels in dairy products and tuna, respectively. Both studies set adsorption and desorption times at 15 and 10 min, respectively. However, herein, adsorption and desorption times of 7.0 and 2.0 min were identified as optimal.

## 4. Conclusions

In this study, a ZIF@PPHF adsorbent was successfully synthesized for the first time and used as a new adsorbent for the microextraction and preconcentration of HIS from food samples. FESEM, BET, and ATR-FTIR analyses confirmed that the prepared adsorbent was successfully synthesized. According to the presented data, the prepared adsorbent particles were 100 nm in size and semi-spherical with certain protrusions. The surface area of the adsorbent was found to be approximately 396.5 m^2^/g, making it well-suited to solid-phase extraction. This study confirmed that ZIF@PPHF adsorbents are effective and well-suited to HIS preconcentration in complex food matrices owing to their high surface area, tunable pore structures, and exceptional adsorption capabilities. The reliability, accuracy, and high adsorption–desorption capacity of the proposed method were validated. Moreover, the reusability of the ZIF adsorbent was demonstrated through multiple adsorption–desorption cycles, with a slight decline in the adsorption efficiency over numerous cycles. Furthermore, the spiked real samples confirmed the validity and accuracy of this method. The percentage mean recoveries ± relative standard deviation (% RSD, *n* = 3) at concentration levels of 5, 10, and 50 mg/L of HIS and using 5 g samples in intra- and inter-day measurements ranged from 97 ± 1.10 to 102.80 ± 0.90 and from 96.40 ± 1.82 to 103.40 ± 0.79, respectively, for the spiked real samples. This versatility will allow for this method’s widespread adoption in food safety monitoring and quality control programs.

## Figures and Tables

**Table 1:** Method validation and quality control programs, including LDR, RE, R^2^, LOD, LOQ, MR ± RSDs, ME, and precision (both intra- and inter-day) of the proposed ZIF@PPHF adsorbent coupled with the HPLC method to quantify histamine in fish and cheese samples.

LDR	RE (R^2^)	LOD	LOQ	Food Samples	Unspiked Results (M ± SD, mg/L)	Spiked Level	Results of Spiked Samples (M ± SD, mg/L)		MR ± RSD (%, *n* = 3)		*ME* (%)
							Intra-Day	Inter-Day	Intra-Day	Inter-Day	
0.05–250	y = 372.73x + 267.78 R^2^ = 0.9989	0.019	0.050	LFF	17.5 ± 0.20	5	22.40 ± 0.26	22.43 ± 0.41	98 ± 1.18	98.60 ± 1.85	96.78
						10	27.20 ± 0.30	27.46 ± 0.40	97 ± 1.10	99.60 ± 1.47	101.84
						50	67.01 ± 0.20	67.57 ± 0.42	99.02 ± 0.29	100.14 ± 0.62	102.20
				HFF	23.66 ± 0.25	5	28.70 ± 0.30	28.60 ± 0.30	102 ± 1.06	98.80 ± 1.04	93.23
						10	33.70 ± 0.45	33.50 ± 0.30	100.40 ± 1.36	98.40 ± 0.89	99.04
						50	72.90 ± 0.8	73.97 ± 0.25	98.48 ± 1.09	100.62 ± 0.34	97.23
				LFC	13.87 ± 0.15	5	18.90 ± 0.20	19.04 ± 0.15	100.60 ± 1.05	103.40 ± 0.79	95.11
						10	23.60 ± 0.62	24.20 ± 0.30	97.30 ± 2.64	103.30 ± 1.23	104.06
						50	62.13 ± 1.20	64.46 ± 0.70	96.52 ± 1.94	101.18 ± 1.08	98.77
				HFC	16.96 ± 0.20	5	22.10 ± 0.20	21.93 ± 0.25	102.80 ± 0.90	99.40 ± 1.14	93.63
						10	26.83 ± 0.30	26.60 ± 0.48	98.70 ± 1.13	96.40 ± 1.82	98.11
						50	67.03 ± 0.30	66.7 ± 0.80	100.14 ± 0.45	99.48 ± 1.19	98.95

**LDR**: linear dynamic range (mg/L); **RE**: regression equation (y, HPLC peak area; x, concentration (mg/L); **R^2^**, determination coefficient; **LOD**: limit of detection (mg/L); **LOQ**: limit of quantification (mg/L); **M ± SD**, mean ± standard deviation (mg/L); **HFF**, high-fat fish; **LFF**, low-fat fish; **HFC**, high-fat cheese; **LFF**, low-fat cheese; **MR ± RSD**: mean recovery ± relative standard deviation (%); ***ME***: matrix effect (%).

**Table 2:** Comparison of the current proposed method with previously reported methods for the preconcentration and determination of histamine.

Analytical Method	Adsorbent	Adsorption Time	Desorption Time	LOD	Type of Samples	References
HPLC-UV	Fe_3_O_4_@MCM-41-SPE nanoparticles	15 min	10 min	0.014 mg/L	Dairy product	[27]
Spectrophotometer	CHI/MMIPs	15 min	10 min	1.5 ng/mL	Tuna fish	[68]
HPLC-UV	PEG-modified halloysite	-	-	0.078 mg/kg	Fish	[42]
HPLC-ELSD	C18 sorbent	-	-	2.1 mg/L	Cheese	[69]
HPLC-UV	C18 sorbent	-	-	0.3 mg/kg	Cheese	[69]
HPLC-UV	ZIF@PPHF	7 min	2 min	0.019 mg/L	Fish and Cheese	This study

HPLC-UV: high-performance liquid chromatography–ultraviolet detector, HPLC-ELSD: high-performance liquid chromatography–evaporative light-scattering detector, Fe_3_O_4_@MCM-41-SPE nanoparticles: magnetic solid-phase extraction based on mesoporous silica-coated iron oxide nanoparticles, CHI/MMIPs: chitosan magnetic molecularly imprinted polymers, PEG-modified halloysite: poly (ethylene glycol) 4-nonylphenyl 3-sulfopropyl ether potassium salt (PEG-NP-S) modified on the inner surface of the lumen of halloysites by ion exchange.

## Data Availability

The original contributions presented in the study are included in the article, further inquiries can be directed to the corresponding author.
